# Calibration Invariance of the MaxEnt Distribution in the Maximum Entropy Principle

**DOI:** 10.3390/e23010096

**Published:** 2021-01-11

**Authors:** Jan Korbel

**Affiliations:** 1 Section for the Science of Complex Systems, Center for Medical Statistics, Informatics, and Intelligent Systems (CeMSIIS), Medical University of Vienna, Spitalgasse 23, 1090 Vienna, Austria; jan.korbel@meduniwien.ac.at; 2 Complexity Science Hub Vienna, Josefstädterstrasse 39, 1080 Vienna, Austria; 3 Faculty of Nuclear Sciences and Physical Engineering, Czech Technical University, 11519 Prague, Czech Republic

**Keywords:** maximum entropy principle, MaxEnt distribution, calibration invariance, Lagrange multipliers

## Abstract

The maximum entropy principle consists of two steps: The first step is to find the distribution which maximizes entropy under given constraints. The second step is to calculate the corresponding thermodynamic quantities. The second part is determined by Lagrange multipliers’ relation to the measurable physical quantities as temperature or Helmholtz free energy/free entropy. We show that for a given MaxEnt distribution, the whole class of entropies and constraints leads to the same distribution but generally different thermodynamics. Two simple classes of transformations that preserve the MaxEnt distributions are studied: The first case is a transform of the entropy to an arbitrary increasing function of that entropy. The second case is the transform of the energetic constraint to a combination of the normalization and energetic constraints. We derive group transformations of the Lagrange multipliers corresponding to these transformations and determine their connections to thermodynamic quantities. For each case, we provide a simple example of this transformation.

## 1. Introduction

The maximum entropy principle (MEP) is one of the most fundamental concepts in equilibrium statistical mechanics. It was originally proposed by Jaynes [1,2] in order to connect information entropy introduced by Shannon and thermodynamic entropy introduced by Clausius, Boltzmann, and Gibbs. Although the MEP was originally introduced for the case of Shannon entropy, with the advent of generalized entropies [3,4,5,6,7,8,9,10,11,12,13,14,15,16,17] the natural effort was to apply the maximum entropy principle beyond the case of Shannon entropy. Another question that arose naturally is whether the MEP can be applied to other than ordinary linear constraints. Examples of the constraints that might be considered in connection with the MEP are *escort constraints* [18,19,20], *Kolmogorov–Nagumo means* [21,22], or more exotic types of constraints [23]. It brought some discussion about the applicability of the principle for the case of generalized entropies [24,25] and nonlinear constraints and its thermodynamic interpretation [26,27,28,29,30]. Indeed, MEP is not the only one extremal principle in statistical physics, let us mention, e.g., the *principle of maximum caliber* [31] which is useful in non-equilibrium physics. In this paper, we stick, however, to MEP, as it is the most widespread principle and the theory of generalized thermostatistics has been mainly focused on MEP. For a recent review of other principles, see also in [32]. For the discussion between entropy arising from information theory and thermodynamics, see in [33]. For the sake of simplicity, let us consider canonical ensemble, i.e., fluctuations in internal energy. For the case of the grand-canonical ensemble, one can obtain similar results to the ones presented in this paper for the case of a chemical potential μ.

In order to grasp the debate about the applicability of the MEP, let us emphasize that the MEP consists of two main parts:

(I) Finding a distribution (*MaxEnt* distribution) that maximizes entropy under given constraints.
(II) Plugging the distribution into the entropic functional and calculating physical quantities as thermodynamic potentials, temperature, or response coefficients (specific heat, compressibility, etc.).

The first part is rather a mathematical procedure of finding a maximum subject to constraints. This is done by the *method of Lagrange multipliers*, by defining a Lagrange function in the form

Lagrangefunction=entropy−(Lagrangemultiplier)·(constraint)

The Lagrange multipliers’ role at this stage is to ensure fulfillment of constraints as they are determined from the set of equations obtained from the maximization of the Lagrange function. This procedure is known in statistics as *Softmax*, a method used to infer distribution from given data. Shore and Johnson [34,35] therefore studied MEP as a statistical inference procedure and established a set of consistency axioms. Shore and Johnson’s work heated a debate about whether MEP for generalized entropies can be also understood as a statistical inference method satisfying the consistency requirements [24,36,37,38,39,40,41]. In [42], it was shown that the class of entropies satisfying the original Shore–Johnson axioms is wider than previously thought. Moreover, in [43], the connection between Shore–Johnson axioms and Shannon–Khinchin axioms was investigated and the equivalence of information theory and statistical inference axiomatics was established.

In the second part, the physical interpretation of entropy starts to arise. Similar to the case of Lagrangian mechanics, where the Lagrangian is the difference between kinetic and potential energy and the Lagrange multipliers play the role of the normal force to the constraints, here the entropy becomes a thermodynamic state variable. For Shannon entropy and linear constraints, the Lagrange multipliers become inverse temperature and free entropy, respectively.

The main aim of this paper is to discuss the relation between points (I) and (II). In the first part, it is possible to find a class of entropic functionals and constraints leading to the same MaxEnt distribution. However, in the second part, different entropy and/or constraints lead to different thermodynamics and different relations between physical quantities and Lagrange multipliers. The two main messages of this paper are listed below.

(i) For each MaxEnt distribution, there exists the whole class of entropies and constraints leading to generally different thermodynamics.
(ii) It is possible to establish transformation relations of Lagrange parameters (and subsequently the thermodynamic quantities) for classes of entropies and constraints giving the same MaxEnt distribution.

We call the latter transformation relation *calibration invariance* of the MaxEnt distribution. A straightforward consequence is that in order to fully determine the statistical properties of a thermal system in equilibrium, it is not enough to measure the statistical distribution of energies.

The rest of the paper is organized as follows. In the next section, we briefly discuss the main aspects of MEP for the case of general entropic functional and general constraints. In the following two sections, we introduce two simple transformations of entropic functional (Section 3) and constraints (Section 4) that lead to the same MaxEnt distribution and derive transformations between the Lagrange multipliers. These transformations form a group. After the general derivation, we provide a few simple examples for each case. The last section is devoted to conclusions.

## 2. Maximum Entropy Principle in Statistical Physics

Maximum entropy principle is the way of obtaining the representing probability distribution from the limited amount of information. Our aim is to find the probability distribution of the system $P={\left\{p_{i}\right\}}_{i=1}^{n}$ under the set of given constraints. In the simplest case, the principle can be formulated as follows.

**Maximum entropy principle:** *Maximize entropy S(P) under the normalization constraint $f_{0}\left(P\right)=0$, and energy constraint $f_{E}\left(P\right)=0$.*

The normalization condition is considered in the regular form, i.e., $f_{0}\left(P\right)=\sum_{i}p_{i}-1=\langle 1\rangle -1$. Moreover, we have a class of constraints, which originally described the average energy of the system. Therefore, we call them *energy constraints*. We consider only one energy constraint, for simplicity, although there can be more constraints, and they do not have to consider only internal energy but also other thermodynamic quantities. In the original formulation, the energy constraint is linear in probabilities, i.e.,

(1) $$f_{E}\left(P\right)=\sum_{i}p_{i}E_{i}-E=\langle E\rangle -E,$$

but it can be generally any nonlinear function of probabilities—escort means provide an example. A large class of energy constraints can be written in a *separable form*, which means that $f_{E}\left(P\right)=E\left(P\right)-E$, i.e., in the form expressing the “expected” internal energy (macroscopic variable) as a function of probability distribution (microscopic variable). This class of constraints plays a dominant role in the thermodynamic systems.

In order to find a solution of the Maximum entropy principle, we use a common method of Lagrange multipliers, which can be done through maximization of Lagrange function:

(2) $$L\left(P;\alpha ,\beta \right)=S\left(P\right)-\alpha f_{0}\left(P\right)-\beta f_{E}\left(P\right)$$

The maximization procedure leads to the set of equations

(3) $$\begin{array}{ccc} \frac{\partial L(P;\alpha ,\beta )}{\partial p_{i}} & = & 0\;\forall \;i\in \{1,\ldots ,n\}\\ \frac{\partial L(P;\alpha ,\beta )}{\partial \alpha } & = & f_{0}\left(P\right)=0\\ \frac{\partial L(P;\alpha ,\beta )}{\partial \beta } & = & f_{E}\left(P\right)=0 \end{array}$$

from which we determine the resulting MaxEnt distribution. In order to obtain a unique solution, we require that the entropic functional should be a Schur-concave symmetric function [42].

As a consequence, we obtain the values of Lagrange multipliers α and β. From the strictly mathematical point of view, Lagrange multipliers are just auxiliary parameters to be solved from the set of Equation (3). However, in physics, Lagrange parameters also have a physical interpretation. In Lagrangian mechanics, Lagrange parameters play the role of normal force to the constraints. Similarly, in ordinary statistical mechanics based on Shannon entropy $H\left(P\right)=-\sum_{i}p_{i}\log p_{i}$ and linear constraints (1), the Lagrange multipliers have the particular physical interpretation:

(4) $$\begin{array}{ccc} \beta & = & \frac{1}{T}\;\left(\mathrm{inverse}\;\mathrm{temperature}\right), \end{array}$$

(5) $$\begin{array}{ccc} \alpha & = & S-\frac{1}{T}E\;\left(\mathrm{free}\;\mathrm{entropy}\right). \end{array}$$

Note that the free entropy is, similarly to Helmholtz free energy, a Legendre transform of entropy w.r.t. internal energy. For the case of ordinary thermodynamics (Shannon entropy and linear constraints), it is equal to the logarithm of the partition function.

This interpretation is valid only in this case. In the case, when we use different entropy functional or different constraints, these relation between Lagrange multipliers and thermodynamic quantities are no longer valid. This is even the case, when the resulting MaxEnt distribution is the same.

The main aim of this paper is to show how the invariance of MaxEnt distribution affects the Lagrange multipliers and their relations to thermodynamic quantities. Let us now solve Equation (3). The first set of equations leads to

(6) $$\frac{\partial S(P)}{\partial p_{i}}-\alpha \frac{\partial f_{0}\left(P\right)}{\partial p_{i}}-\beta \frac{\partial f_{E}\left(P\right)}{\partial p_{i}}=0.$$

Let us assume the normalization in the usual way which leads to $\frac{\partial f_{0}\left(P\right)}{\partial p_{i}}=1$. Moreover, let us consider separable energy constraint, so $\frac{\partial f_{E}\left(P\right)}{\partial p_{i}}=\frac{\partial E(P)}{\partial p_{i}}$. The resulting probability distribution can be expressed as

(7) $$p_{i}^{⋆}={\frac{\partial S}{\partial p_{i}}}^{\left(-1\right)}\left[\alpha +\beta \frac{\partial E(P)}{\partial p_{i}}\right].$$

where ${}^{\left(-1\right)}$ denotes inverse function of $\partial S/\partial p_{i}$ (provided it exists and is unique). We can express α by multiplying the equation by $p_{i}$ and summing over *i*, which leads to

(8) $$\alpha =\langle \nabla_{P}S\left(P\right)\rangle -\beta \langle \nabla_{P}E\left(P\right)\rangle$$

where $\langle X\rangle =\sum_{i}x_{i}p_{i}$ and $\nabla_{P}=\left(\frac{\partial }{\partial p_{1}},\ldots ,\frac{\partial }{\partial p_{n}}\right)$. By plugging back to the previous equation, we can get β as

(9) $$\beta =\frac{\Delta_{i}\left(\nabla S\left(P\right)\right)}{\Delta_{i}\left(\nabla E\left(P\right)\right)}$$

where $\Delta_{i}\left(X\right)=x_{i}-\langle X\rangle$ is the difference from the average.

The solution of Equation (3) depends on the internal energy *E*. However, in thermodynamics it is natural to invert the relation β=β(E) and express the relevant quantities in terms of β, so E=E(β). With that, we can calculate dependence of entropy on β:

(10) $$\frac{\partial S}{\partial \beta }=\sum_{i}\frac{\partial S}{\partial p_{i}}\frac{\partial p_{i}}{\partial \beta }=\sum_{i}\left(\alpha +\beta \frac{\partial E(P)}{\partial p_{i}}\right)\frac{\partial p_{i}}{\partial \beta }=\beta \sum_{i}\frac{\partial f_{E}}{\partial p_{i}}\frac{\partial p_{i}}{\partial \beta }=\beta \left(-\frac{\partial f_{E}}{\partial E}\frac{\partial E}{\partial \beta }\right)$$

For separable energy constraints, $\frac{\partial f_{E}}{\partial E}=-1$, so we obtain the well-known relation

(11) $$\frac{\partial S}{\partial \beta }=\beta \frac{\partial E}{\partial \beta }\Rightarrow \beta =\frac{\partial S}{\partial E}.$$

Let us now define the Legendre conjugate of entropy called *free entropy* (also called Jaynes parameter [44] or Massieu function [45]):

(12) $$\psi =S-\frac{\partial S}{\partial E}E=S-\beta E$$

Free entropy is connected to Helmholtz free energy as ψ=−βF. The difference between α and ψ can be expressed as

(13) $$\psi -\alpha =\left(S-\langle \nabla_{P}S\rangle \right)-\beta \left(E-\langle \nabla_{P}E\rangle \right)$$

Therefore, we can understand the difference ψ−α as the Legendre transform of ψ with respect to *P*. From this, we see that the difference between ψ and α is a constant (not depending on thermodynamic quantities), if two independent conditions are fulfilled, i.e., $E=\langle \nabla_{P}E\left(P\right)\rangle$ and $S=\langle \nabla_{P}S\rangle +a$. The former constraint leads to linear energy constraints, while the latter one leads to the the conclusion that the entropy must be in trace form $S\left(P\right)=\sum_{i}g\left(p_{i}\right)$. Moreover, the function *g* has to fulfill the following equation,

(14) $$g\left(x\right)-ax=xg^{\prime }\left(x\right)$$

leading to g(x)=−axlog(x)+bx which is equivalent to Shannon entropy.

In the next sections, we will explore how the transformation of the entropy and the energy constraint that leaves the MaxEnt distribution invariant affects the Lagrange multipliers and their relation to thermodynamic quantities.

## 3. Calibration Invariance of MaxEnt Distribution with Entropy Transformation

The simplest transformation of Lagrange functional that leaves the MaxEnt distribution invariant is to consider an arbitrary increasing function of entropy, i.e., we replace S(P) by c(S(P)), where $c^{\prime }\left(x\right)>0$. Let us note that this transform preserves the uniqueness of the MEP because it is easy to show that if S(P) is Schur-concave, c(S(P)) is also Schur-concave [42] which is a sufficient condition for uniqueness of the MaxEnt distribution.

In this case, the Lagrange equations are adjusted as follows,

(15) $$c^{\prime }\left(S\left(P\right)\right)\frac{\partial S(P)}{\partial p_{i}}-\alpha_{c}\frac{\partial f_{0}\left(P\right)}{\partial p_{i}}-\beta_{c}\frac{\partial E(P)}{\partial p_{i}}=0$$

leading to

(16) $$\alpha_{c}=c^{\prime }\left(S\left(P\right)\right)\langle \nabla_{P}S\left(P\right)\rangle -\beta_{c}\langle \nabla_{P}E\left(P\right)\rangle$$

and

(17) $$\beta_{c}=c^{\prime }\left(S\left(P\right)\right)\frac{\Delta_{i}\left(\nabla_{P}S\left(P\right)\right)}{\Delta_{i}\left(\nabla_{P}E\left(P\right)\right)}$$

so we get that the function *c* causes *rescaling* of α and β, so

(18) $$\begin{array}{c} \alpha_{c}=c^{\prime }\left(S\left(P\right)\right)\;\alpha \end{array}$$

(19) $$\begin{array}{c} \beta_{c}=c^{\prime }\left(S\left(P\right)\right)\;\beta \end{array}$$

while its ratio remains unchanged, i.e., $\alpha_{c}/\beta_{c}=\alpha /\beta$. Actually, the set of increasing functions conform a group of Lagrange multipliers, because it is easy to show that the Lagrange parameters related to the entropy $c_{1}(c_{2}\left(S\left(P\right)\right)$

(20) $$\beta_{c_{1}\circ c_{2}}=c_{1}^{\prime }(c_{2}\left(S\left(P\right)\right)\cdot c_{2}^{\prime }\left(S\left(P\right)\right)\;\beta =c_{1}^{\prime }(c_{2}\left(S\left(P\right)\right)\beta_{c_{2}}$$

which can be described as the group operation $\left(c_{1}\circ c_{2}\right)\mapsto c_{1}^{\prime }\left(c_{2}\right)\cdot c_{2}^{\prime }$.

An important property of this transformation is that it changes the extensive–intensive duality of the conjugated pair of thermodynamic variables and the respective forces while it maintains the distribution. Notably, by changing the entropic functional from extensive (i.e., S(n)∼U(n)) to non-extensive, it changes β from intensive (i.e., size-independent, at least in the thermodynamic limit) to non-intensive, i.e., explicitly size-dependent. This point has been discussed in connection with *q*-non-extensive statistical physics of [29,30] and the relation to the zeroth law of thermodynamics was shown in [46]. As one can see from the example below, although Rényi entropy and Tsallis entropy have the same maximizer, the corresponding thermodynamics is different. While Rényi entropy is additive (and therefore extensive for systems where U(n)∼n) and the temperature is intensive, Tsallis entropy is non-extensive, and the corresponding temperature explicitly depends on the size of the system.

Let us finally mention that the difference between free entropy and Lagrange parameter α transforms as

(21) $$\psi_{c}-\alpha_{c}=(c\left(S\right)-c^{\prime }\left(S\right)\langle \nabla_{P}S\left(P\right)\rangle -c^{\prime }\left(S\right)\beta \left(E-\langle \nabla_{P}E\left(P\right)\rangle \right)=c^{\prime }\left(S\right)\left(\psi -\alpha \right)+\left(c\left(S\right)-c^{\prime }\left(S\right)\cdot S\right).$$

While free entropy and other thermodynamic potentials are transformed, the heat change remains invariant under this transformation:

(22) $$đQ_{c}=T_{c}d\;c\left(S\right)=\frac{T}{c^{\prime }\left(S\right)}c^{\prime }\left(S\right)dS=TdS=đQ.$$

**Example** **1.** *We exemplify the calibration invariance on two popular examples of closely related entropies.*

- **Rényi entropy and Tsallis entropy** *: Two most famous examples of generalized entropies are Rényi entropy $R_{q}\left(P\right)=\frac{1}{1-q}\ln\left(\sum_{i}p_{i}^{q}\right)$ and Tsallis entropy $S_{q}\left(P\right)=\frac{1}{1-q}\left(\sum_{i}p_{i}^{q}-1\right)$. Their relation can be expressed as*
  (23) $$R_{q}\left(P\right)=c_{q}\left(S_{q}\left(P\right)\right)=\frac{1}{1-q}\ln\left[\left(1-q\right)S_{q}\left(P\right)+1\right]$$
*and therefore we obtain that*
  (24) $$c_{q}^{\prime }\left(S_{q}\left(P\right)\right)=\frac{1}{1+\left(1-q\right)S_{q}}=\frac{1}{\sum_{i}p_{i}^{q}}.$$
*The difference between free entropy and α can be obtained as*
  (25) $$\psi_{R}-\alpha_{R}=\frac{1}{\sum_{i}p_{i}^{q}}\left(\psi_{S}-\alpha_{S}\right)+\left(R_{q}\left(P\right)-\frac{S_{q}\left(P\right)}{\sum_{i}p_{i}^{q}}\right).$$
*One can therefore see that even though Rényi and Tsallis entropy lead to the same MaxEnt distribution, their thermodynamic quantities, such as temperature or free entropy, are different. Whether the system follows Rényi or Tsallis entropy depends on additional facts, as e.g., (non)-extensitivity and (non)-intensivity of thermodynamic quantities.*
- **Shannon entropy and Entropy power** *: A similar example is provided with Shannon entropy $H\left(P\right)=\sum_{i}p_{i}\ln1/p_{i}$ and entropy power $P\left(P\right)=\prod_{i}{\left(1/p_{i}\right)}^{p_{i}}$. The relation between them is simply*
  (26) H(P)=c(P(P))=log(P(P)),
*so we obtain that*
  (27) $$c^{\prime }\left(P\left(P\right)\right)=1/\left(P\left(P\right)\right)=\exp\left(-H\left(P\right)\right).$$
*For the difference between free entropy and α, we obtain that*
  (28) $$0=\psi_{H}-\alpha_{H}=\frac{1}{P(P)}\left(\psi_{P}-\alpha_{P}\right)+\left(H(P)-1\right)$$
*from which we get that*
  (29) $$\psi_{P}-\alpha_{P}=P\left(P\right)\left(1-\log P(P)\right).$$
*Therefore, we see that even that the MaxEnt distribution remains unchanged, the relation between α and free energy is different.*

## 4. Calibration Invariance of MaxEnt Distribution with Constraints Transformation

Similarly, one can uncover the invariance of the MaxEnt distribution when the constraints are transformed in a certain way. Generally, if two sets of constraints define the same domain, the resulting Maximum entropy principle should lead to equivalent results. We will not be so general, but we focus on a specific situation, which might be quite interesting for thermodynamic applications. Let us remind two conditions, which we assume: normalization $f_{0}\left(P\right)=0$ and energy constraint $f_{E}\left(P\right)=0$. Let us investigate the latter. Similarly to the previous case, it is possible to take any function *g* of $f_{E}\left(P\right)$, for which g(y)=0 if y=0. More generally, we can also take into account the normalization constraint and replace the original energy condition by

(30) $$g(f_{0}\left(P\right),f_{E}\left(P\right))=0$$

for any g(x,y), for which g(x,y)=0⇒y=0. Let us investigate the Maximum entropy principle for this case. We can express the Lagrange function as

(31) $$L\left(P\right)=S\left(P\right)-\alpha_{g}f_{0}\left(P\right)-\beta_{g}g\left(f_{0}\left(P\right),f_{E}\left(P\right)\right)$$

which leads to a set of equations

(32) $$\frac{\partial S(P)}{\partial p_{i}}-\alpha_{g}\frac{\partial f_{0}\left(P\right)}{\partial p_{i}}-\beta_{g}\left[G^{\left(1,0\right)}\frac{\partial f_{0}\left(P\right)}{\partial p_{i}}+G^{\left(0,1\right)}\frac{\partial E(P)}{\partial p_{i}}\right]=0$$

where $G^{\left(1,0\right)}=\frac{\partial g(x,y)}{\partial x}{|}_{\left(0,0\right)}$ and $G^{\left(0,1\right)}=\frac{\partial g(y,x)}{\partial x}{|}_{\left(0,0\right)}$. We take again into account that $\frac{\partial f_{0}\left(P\right)}{\partial p_{i}}=1$, multiply the equations by $p_{i}$ and some over *i*. This gives us

(33) $$\alpha_{g}=\langle \nabla_{P}S\left(P\right)\rangle -\beta_{g}\left[G^{\left(1,0\right)}+G^{\left(0,1\right)}\langle \nabla_{P}E\left(P\right)\rangle \right]\;.$$

By plugging $\alpha_{g}$ back, we end with relation for $\beta_{g}$:

(34) $$\beta_{g}=\frac{1}{G^{\left(0,1\right)}}\frac{\Delta_{i}\left(\nabla_{P}S\left(P\right)\right)}{\Delta_{i}\left(\nabla_{P}E\left(P\right)\right)}\;.$$

For $\alpha_{g}$ we end with

(35) $$\alpha_{g}=\langle \nabla_{P}S\left(P\right)\rangle -\frac{\Delta_{i}\left(\nabla_{P}S\left(P\right)\right)}{\Delta_{i}\left(\nabla_{P}E\left(P\right)\right)}\langle \nabla f_{E}\left(P\right)\rangle \left[1+\frac{G^{\left(1,0\right)}}{G^{\left(0,1\right)}}\frac{1}{\langle \nabla_{P}E\left(P\right)\rangle }\right]\;.$$

Thus, we end again with rescaling of $\alpha_{g}$ and $\beta_{g}$, which reads

(36) $$\begin{array}{c} \alpha_{g}\left(\alpha ,\beta \right)=\alpha -\frac{G^{\left(1,0\right)}}{G^{\left(0,1\right)}}\beta \;, \end{array}$$

(37) $$\begin{array}{c} \beta_{g}\left(\beta \right)=\frac{\beta }{G^{\left(0,1\right)}}\;. \end{array}$$

The ratio of Lagrange multipliers is also transformed, so we get

(38) $$\frac{\alpha_{g}}{\beta_{g}}=G^{\left(0,1\right)}\frac{\alpha }{\beta }-G^{\left(1,0\right)}.$$

Again, the set of all functions fulfilling the aforementioned condition conform a group. The group operation can be described by the relation between coefficients $G^{\left(1,0\right)}$ and $G^{\left(0,1\right)}$ for the composite function $g\left(x,y\right)=g_{1}\left(x,g_{2}\left(x,y\right)\right)$. We obtain that

(39) $$\begin{array}{ccc} G^{\left(1,0\right)} & = & G_{1}^{\left(1,0\right)}+G_{1}^{\left(0,1\right)}G_{2}^{\left(1,0\right)} \end{array}$$

(40) $$\begin{array}{ccc} G^{\left(0,1\right)} & = & G_{1}^{\left(0,1\right)}G_{2}^{\left(0,1\right)} \end{array}$$

which leads to group relations

(41) $$\begin{array}{ccc} \alpha_{g}\left(\alpha ,\beta \right) & = & \alpha_{g_{1}}\left(\alpha_{g_{2}}\left(\alpha ,\beta \right),\beta_{g_{2}}\left(\beta \right)\right)-\frac{G_{1}^{\left(1,0\right)}}{G_{1}^{\left(0,1\right)}}\beta_{g_{2}}\left(\beta \right) \end{array}$$

(42) $$\begin{array}{ccc} \beta_{g}\left(\beta \right) & = & \frac{\beta_{g_{2}}\left(\beta \right)}{G_{1}^{\left(0,1\right)}}. \end{array}$$

**Example** **2.** *Here we mention two simple examples of the aforementioned transformation.*

- **Energy shift:** *Under this scheme, we can assume the constant shift in the energy spectrum. Let us rewrite the constraint f(P) in the following form,*
  (43) $$f_{E}\left(P\right)=\sum p_{i}E_{i}-E=\sum p_{i}\left(E_{i}-E^{\prime }\right)-\left(E-E^{\prime }\right)$$
*which allows us to identify the function g(x,y) as*
  (44) $$g\left(x,y\right)=y-E^{\prime }x+E^{\prime }$$
  *We obtain $G^{\left(1,0\right)}=-E^{\prime }$ and $G^{\left(0,1\right)}=1$, which means that $\alpha^{\prime }=\alpha -\beta E^{\prime }$.*
- **Latent escort means:** *Apart from linear means, it is possible to use some generalized approaches. One of these examples is provided by so-called escort mean:*
  (45) $$E_{q}={\langle E\rangle }_{q}=\frac{\sum_{i}p_{i}^{q}E_{i}}{\sum_{i}p_{i}^{q}}$$
  *which for q=1 becomes an ordinary linear mean, when $P={\left\{p_{i}\right\}}_{i=1}^{n}$ are normalized to one. When we use this class of means in the Maximum entropy principle, the normalization is enforced by the normalization condition $f_{0}\left(P\right)=0$, therefore for q=1 we obtain the same results. Nevertheless, by taking q=1 for the results with escort distribution, the energy constraint is actually expressed as*
  (46) $$\frac{\sum p_{i}E_{i}}{\sum p_{i}}-E$$
  *can be understood in the same way as considered before in this section, i.e., as a combination of a normalization constraint and energy constraint. In this case the function g has the following form,*
  (47) $$g\left(x,y\right)=\frac{y+E}{x+1}-E.$$
*Therefore, we obtain that $G^{\left(1,0\right)}=-E$ and $G^{\left(0,1\right)}=1$, which correspond to the previous example for $E^{\prime }=E$. Therefore, the latent energy mean can be understood in terms of MaxEnt procedure as the shift of the energy spectrum by its average energy.*

## 5. Conclusions

In this paper, we have discussed the calibration invariance of MEP, which means that for a given MaxEnt distribution, there exists a whole class of entropies and constraints that lead to different thermodynamics (Thermodynamic quantities and response coefficients generally have different behavior. For example, from intensive temperature we can obtain temperature that explicitly depends on the size of the system). We have stressed that the MEP procedure consists of two parts, where the first part, consisting of determining the MaxEnt distribution, is rather a mathematical tool, while the second part, making connection between Lagrange multipliers and thermodynamic quantities, is a specific for application of MEP in statistical physics. Indeed, the paper does not cover all possible transformations leading to the same MaxEnt distribution (let us mention, at least, the additive duality of Tsallis entropy, where maximizing $S_{2-q}$ with linear constraint leads to the same result as maximizing $S_{q}$ with escort constraints [47]). The main lesson of this paper is that in order to fully determine a thermal system in equilibrium, we need to measure not only probability distribution, but also all relevant thermodynamic quantities (as entropy). Moreover, the transformation between Lagrange parameters and its connection to thermodynamic potentials can be useful in situations when one is not certain about the exact form of entropy.
